# Long-term Outcomes 18 Years after the Arthroscopic Fixation of a Scapular Articular Fracture: A Case Report

**DOI:** 10.1055/s-0044-1790595

**Published:** 2024-12-27

**Authors:** Carlos Henrique Ramos, Rafaella Monteiro Barbosa, Yasmin Netto Costa Gomes, Ana Luisa Garcia de Paula, Laysla Danyela Coradin Gulicz

**Affiliations:** 1 Hospital de Clínicas, Universidade Federal do Paraná, Curitiba, PR, Brasil; 2 Faculdade Pequeno Príncipe, Curitiba, PR, Brasil; 3 Faculdade Evangélica Mackenzie do Paraná, Curitiba, PR, Brasil; 4 Residência Médica em Ortopedia e Traumatologia, Hospital XV, Curitiba, PR, Brasil

**Keywords:** arthroscopy, fracture fixation, glenoid cavity, scapula, shoulder fractures

## Abstract

Reduction and fixation of glenoid cavity fractures using arthroscopy cause little surgical trauma, allowing the complementary diagnosis and treatment of potentially associated injuries (either capsular, ligamentous or tendon lesions) with promising outcomes. The authors report a case of Ideberg type III glenoid fracture with a distal clavicle fracture which underwent percutaneous reduction and bone fixation (with Kirschner wires) using an arthroscopic technique. We describe the procedure and the outcomes after 18 years of follow-up. The clinical assessment included the functional University of California at Los Angeles (UCLA) score criteria and radiographic studies. The result was excellent/satisfactory, with the patient asymptomatic over time and without relevant radiographic changes. Although the management of glenoid fractures by arthroscopy remains evolving, it is a good treatment alternative to the open approach, especially in less complex fractures.

## Introduction

Scapular fractures account for approximately 1% of all fractures, affecting the articular surface in 10% of cases. 1 Percutaneous reduction and fixation of these fractures using arthroscopy provide articular visualization with precise reduction, diagnosis, and treatment of any associated injuries (either capsular, ligamentous, or tendon lesions), lower surgical trauma reduced blood loss, and better aesthetic results. 2 Studies demonstrating good outcomes are promising, but most have short or medium-term follow-up. 3 4 This paper aims to report one case of displaced glenoid fracture submitted to percutaneous reduction and fixation with arthroscopy and demonstrate long-term outcomes (18 years).

## Case Report

The ethics committee of our institution approved this case report under number CAAE 52798421.4.0000.0020.

**Fig. 1:**
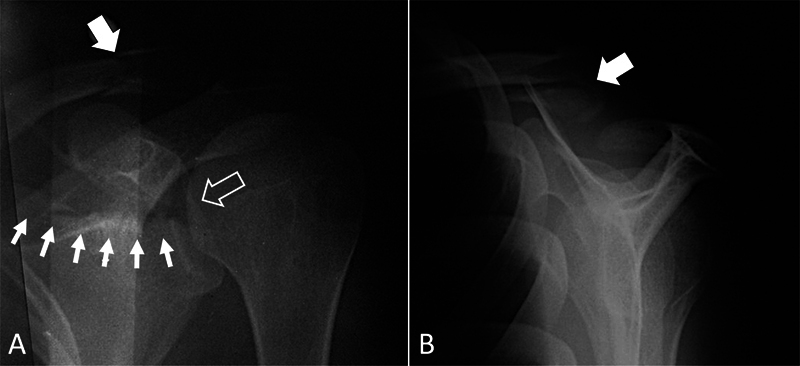
Radiographs of the left shoulder. The anteroposterior view (A) demonstrates the transverse and displaced scapular fracture involving the glenoid cavity and extending to the base of the coracoid process, classified as Ideberg type III (small arrows); detail of the joint displacement (large empty arrow); detail of the fracture in the distal clavicle (large solid arrow). The lateral view (B) details the displacement in the distal clavicular fracture (large solid arrow). Source: authors' archive.

A 27-year-old male, right-handed patient, working as a systems analyst, suffered trauma to the left shoulder after falling off a motorcycle in January 2005. On physical examination, he presented edema and pain in the left scapular and clavicular regions with functional loss of the same shoulder. The patient presented preserved neurological function and perfusion of the upper limb and no other systemic injuries. A simple radiograph showed a scapular fracture involving the glenoid cavity, transverse and displaced, extending to the base of the coracoid process, classified as type III by the Ideberg criteria. 4 On the same side, there was a displaced distal clavicular fracture ( Fig. 1 ). The patient underwent surgical treatment on the second day after the trauma, with reduction and percutaneous fixation of the glenoid and clavicle fractures assisted by arthroscopy.

### Surgical Technique and Outcome

**Fig. 2:**
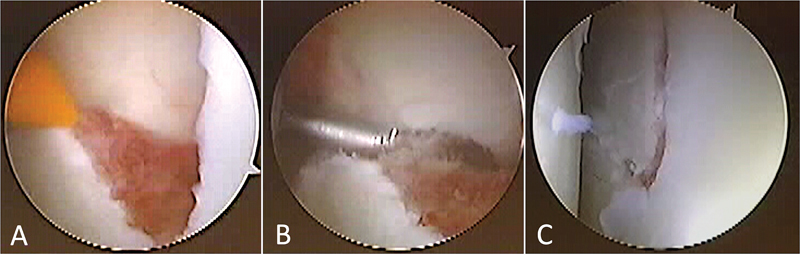
Photographs of the arthroscopic surgical procedure. (A) Detail of the glenoid joint displacement after debridement of the fracture hematoma. (B) Detail of the reduction with a dissector-type instrument. (C) Detail of the final joint reduction. Source: authors' archive.

**Fig. 3:**
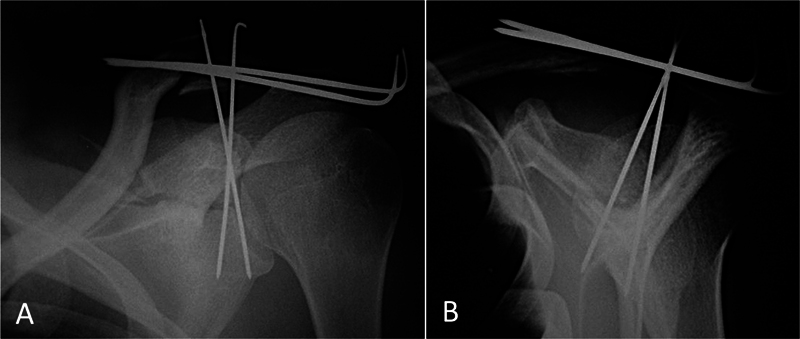
Radiographs of the left shoulder demonstrating immediate postoperative follow-up in anteroposterior (A) and lateral (B) views after percutaneous fixation of the scapula and distal clavicle fractures with Kirschner wires. Source: authors' archive.

**Fig. 4:**
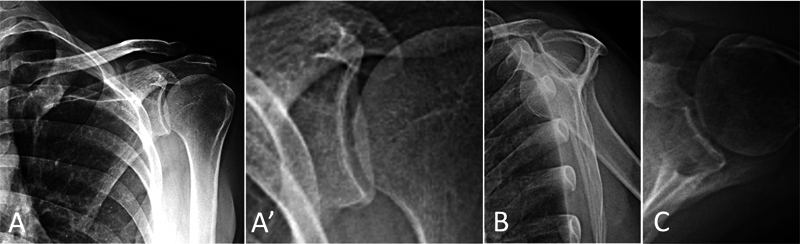
Photographs showing details of the clinical examination at 18 years of postoperative follow-up, with symmetrical shoulder joint range, trophism, and satisfactory aesthetic appearance. (A) Anterior flexion range. (B) Medial rotation range. (C and D) Trophism and posterior and anterior aesthetic appearance of the shoulders, respectively; (E) Lateral rotation range. Source: authors' archive.

**Fig. 5:**
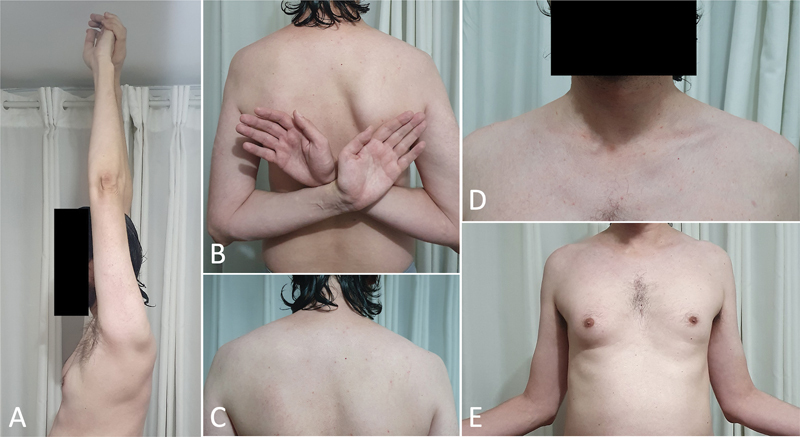
Radiographs of the left shoulder in anteroposterior (A), lateral (B), and axillary (C) views 18 years after the initial surgical treatment, demonstrating good appearance and no relevant changes. (A) Details of the glenoid contour and joint space. Source: authors' archive.

We put the patient in the supine “beach chair” position under general anesthesia, interscalene block, and the image intensifier in place. After arthroscopic joint inspection to rule out associated injuries, we debrided and reduced the fracture site using a dissector-type instrument under simultaneous radioscopic control ( Fig. 2 ). After satisfactory reduction, we performed bone fixation with two percutaneous 1.5 mm Kirschner (K) wires inserted through the superior surface of the glenoid. Lastly, we performed the supplementary percutaneous fixation of the clavicle fracture. Postoperative radiographs confirmed good reduction and K wire positioning ( Fig. 3 ). We immobilized the shoulder with a simple sling for four weeks and instructed the patient to start elbow, wrist, and hand exercises immediately. Gain in joint range of motion and muscle strength began after 3 and 6 weeks, respectively. We removed the K wires after radiographic confirmation of the consolidation six weeks after the index procedure. The outcome assessment included clinical examination, radiographs, and the University of California at Los Angeles (UCLA) score criteria. 5 In follow-up visits 1 and 18 years after surgery, the patient was asymptomatic, with a symmetrical shoulder range of motion (UCLA score of 35, i.e., satisfactory/excellent) and no relevant radiographic changes ( Figs. 4 and 5 ).

## Discussion

The current management of scapular fractures involving the glenoid cavity and displacements higher than 3 to 5 mm recommends surgical reduction and osteosynthesis. 4 Traditional surgical approaches use arthrotomy, which allows good visualization, reduction, and fixation of the fracture for early mobilization. Although effective, they usually involve extensive accesses, with significant surgical morbidity. 1 2 4 The arthroscopic alternative introduced by Carro et al. 6 in 1999 to treat glenoid rim fractures is an efficient option and causes less surgical trauma. Other authors have demonstrated the technique for more complex fractures, including recommendations and tips. 2 4 Most publications refer to the fixation of Ideberg type III fractures, similar to our case, demonstrating satisfactory outcomes, no complications, and the advantages previously mentioned. Disadvantages include the need for local hospital structure, learning curve, and surgeon skills. Regarding the technique, most authors recommend the beach chair position due to the eventual need for conversion to open surgery. 2 3 7 In 2016, Park 8 reported using cannulated screws as an easier alternative, considering that the upper fragment is often single (type III fractures). Their insertion in an anterograde manner through the superior Neviaser portal offers a low risk of nerve and vessel injury. 9 Guides for knee surgery can facilitate wire insertion. 3 Bonczek et al. 7 suggested another technical detail, i.e., using the coracoid process as a joystick for indirect reduction. Associated injuries may require fixation, such as the distal clavicle presented by our patient. 10 The literature is scarce, with limited experience and short- to medium-term outcomes. Yang et al. 3 reported the largest experience, with 18 cases and a mean follow-up of 2 to 5 years. Although this report refers to a single case, we demonstrated the longest follow-up time according to the literature, and our patient is asymptomatic and has no radiographic signs of glenohumeral osteoarthritis for this fracture profile. While the arthroscopic management of glenoid fractures is still evolving and requires studies with longer case series and follow-up time, it is a good treatment alternative to the open approach, especially in less complex fractures.
