# Supplementary material for: Long-term Outcomes 18 Years after the Arthroscopic Fixation of a Scapular Articular Fracture: A Case Report
Source: Rev Bras Ortop (Sao Paulo). 2024 Dec 27;59(Suppl 2):e194–8. doi: 10.1055/s-0044-1790595 (PMC11679628; doi:10.1055/s-0044-1790595)
Supplement: Related texts

# Research article: Resultados a longo prazo após 18 anos de fixação artroscópica de fratura articular da escápula: Relato de caso

**Keywords:** artroscopia, cavidade glenoide, escápula, fixação de fratura, fraturas do ombro

## Abstract

A redução e fixação das fraturas articulares da cavidade glenoidal pela técnica artroscópica causam pouco trauma cirúrgico, possibilitando o diagnóstico complementar e tratamento de eventuais lesões associadas (capsulares, ligamentares e/ou tendinosas), sendo demonstrados resultados promissores até o momento. Os autores relatam um caso de fratura glenoidal tipo III de Ideberg, associada à fratura da clavícula distal, submetida a redução e fixação óssea percutânea (fios de K) assistida pela técnica artroscópica. Descrevem a técnica e o resultado após 18 anos de acompanhamento, com a avaliação clínica realizada segundo critérios funcionais do *score* University of California at Los Angeles (UCLA) e o estudo radiográfico. O resultado foi considerado excelente/satisfatório, com paciente assintomático ao longo do tempo e ausência de alterações radiográficas relevantes. Apesar do manejo de fraturas da glenoide pela artroscopia ainda estar em evolução, apresenta-se como boa alternativa de tratamento em relação à abordagem aberta, especialmente nas fraturas de menor complexidade.

## Introdução

As fraturas da escápula são responsáveis por aproximadamente 1% de todas as fraturas, acometendo a superfície articular em 10% dos casos. 1 A redução e fixação percutânea pela técnica artroscópica destas fraturas proporcionam visão articular com redução precisa, diagnóstico e tratamento de eventuais lesões associadas (capsulares, ligamentares e/ou tendinosas), menor trauma cirúrgico e perda sanguínea, além do melhor resultado estético. 2 Os trabalhos demonstrando bons resultados até o momento são promissores, porém a maioria apresenta acompanhamento de curto ou médio prazo. 3 4 O objetivo deste artigo é relatar um caso de fratura desviada da glenóide submetida à redução e fixação percutânea com auxílio da artroscopia, demonstrando resultado a longo prazo (18 anos).

## Relato de Caso

O referido Relato de Caso teve a aprovação do comitê de ética da nossa instituição sob o número CAAE: 52798421.4.0000.0020.

Paciente do sexo masculino, 27 anos, dominante à direita, analista de sistemas, teve trauma no ombro esquerdo por queda de motocicleta em janeiro de 2005. Ao exame físico apresentava edema e dor nas regiões escapular e clavicular esquerdos, associados à perda funcional do mesmo ombro. A função neurológica e perfusão do membro superior estavam preservadas, sem outras lesões sistêmicas. A radiografia simples demonstrava fratura da escápula, envolvendo a cavidade glenoidal, transversa e desviada, estendendo-se à base do processo coracoide, classificada como tipo III pelos critérios de Ideberg. 4 No mesmo lado havia fratura da clavícula distal desviada ( Fig. 1 ). O paciente foi submetido ao tratamento cirúrgico no segundo dia após o trauma, com redução e fixação percutânea das fraturas da glenoide e clavícula, assistida com artroscopia.

**Fig. 1:** Radiografias do ombro esquerdo, com incidência anteroposterior (A) demonstrando a fratura da escápula envolvendo a cavidade glenoidal, transversa e desviada, estendendo-se à base do processo coracoide, classificada como tipo III de Ideberg (setas menores); detalhe do desvio articular (seta maior vazia); detalhe da fratura na clavícula distal (seta maior cheia). Incidência em perfil (B) demonstrando detalhe do desvio na fratura da clavícula distal (seta maior cheia). Fonte: arquivo dos autores.

## Técnica Cirúrgica e Resultado

O paciente foi posicionado em decúbito dorsal tipo “cadeira de praia,” sob anestesia geral e bloqueio interescalênico, com intensificador de imagens posicionado. Após inspeção articular artroscópica, descartando-se lesões associadas, o foco da fratura foi debridado e reduzido com ajuda de instrumento tipo descolador, sob controle radioscópico simultâneo ( Fig. 2 ). Após redução satisfatória, a fixação óssea foi realizada com 2 fios de Kirschners (K) 1.5 mm percutâneos, inseridos pela face superior da glenoide. Fixação percutânea complementar da fratura da clavícula foi realizada ao final. Radiografias pós-operatórias (PO) confirmaram boa redução e posição dos fios de K ( Fig. 3 ). O ombro foi imobilizado com tipóia simples durante 4 semanas, iniciando-se exercícios imediatos para cotovelo, punho e mão. Ganho de amplitude articular e força muscular foram iniciados após 3 e 6 semanas respectivamente. Os fios de K foram retirados após confirmação radiográfica da consolidação (6 semanas). O resultado foi avaliado utilizando-se o exame clínico, radiografias e os critérios do *Score UCLA* . 5 Nas revisões após 1 e 18 anos, o paciente apresentava-se assintomático, com amplitude articular simétrica dos ombros ( *UCLA* 35 - satisfatório/excelente) e sem alterações radiográficas relevantes ( Figs. 4 e 5 ).

**Fig. 2:** Fotografias demonstrando imagens do procedimento cirúrgico assistido pela artroscopia; A: detalhe do desvio articular glenoidal após debridamento do hematoma fraturário; B: detalhe da redução com auxílio de instrumento tipo descolador; C: detalhe da redução articular final. Fonte: arquivo dos autores.

**Fig. 3:** Radiografias do ombro esquerdo demonstrando controle pós-operatório imediato nas incidências anteroposterior (A) e perfil (B), após fixação percutânea das fraturas da escápula e clavícula distal com fios de K. Fonte: arquivo dos autores.

**Fig. 4:** Fotografias demonstrando detalhes do exame clínico após 18 anos de seguimento pós-operatório, com amplitude articular simétrica dos ombros, trofismo e aspecto estético satisfatórios; A: amplitude da flexão anterior; B: amplitude da rotação medial; C e D: trofismo e aspecto estético posterior e anterior dos ombros, respectivamente; E: amplitude da rotação lateral. Fonte: arquivo dos autores.

**Fig. 5:** Radiografias do ombro esquerdo em incidências anteroposterior (A), perfil (B) e axilar (C) após 18 anos do tratamento cirúrgico inicial, demonstrando bom aspecto, sem alterações relevantes; A': detalhes do contorno glenoidal e espaço articular. Fonte: arquivo dos autores.

## Discussão

No manejo atual das fraturas da escápula que envolvem a cavidade glenoidal e desvios maiores que 3–5mm, recomenda-se redução cirúrgica e osteossíntese. 4 Nas abordagens cirúrgicas tradicionais utiliza-se a artrotomia, que permite boa visualização, redução e fixação da fratura, com objetivo da mobilização precoce. Apesar de eficazes, normalmente são acessos extensos, com morbidade cirúrgica relevante. 1 2 4 A alternativa artroscópica, descrita inicialmente por Carro et al. 6 em 1999 para o tratamento das fraturas do rebordo glenoidal, foi descrita como opção eficiente, causando menor trauma cirúrgico. Outros autores demonstraram a técnica para fraturas mais complexas, incluindo recomendações e dicas. 2 4 A maioria das publicações se refere à fixação das fraturas tipo III de Ideberg, semelhantes ao nosso caso, demonstrando resultados satisfatórios, sem complicações, associadas às vantagens relatadas acima. Como desvantagens, há a necessidade da estrutura hospitalar local, curva de aprendizado e habilidade do cirurgião. Quanto à técnica, a maioria dos autores recomenda realizá-la na posição tipo cadeira de praia, pela eventual necessidade da conversão para cirurgia aberta. 2 3 7 Park 8 em 2016, relatou o uso de parafusos canulados como alternativa mais fácil, considerando que o fragmento superior geralmente é único (fraturas tipo III). Sua inserção de modo anterógrado pelo portal superior de Neviaser, oferece baixo risco para lesão de nervos e vasos. 9 Guias utilizados nas cirurgias de joelho podem facilitar a inserção dos fios. 3 Outro detalhe técnico, sugerido por Bonczek et al., 7 foi utilizar o processo coracoide como *joystick* para redução indireta. Lesões associadas podem necessitar fixação, como da clavícula distal em nosso caso. 10 A maioria das referências demonstra relatos de poucos casos, com experiência reduzida e resultados de curto a médio prazo. A maior experiência foi publicada por Yang et al., 3 com 18 casos e acompanhamento médio de 2 a 5 anos. Apesar de 1 caso, demonstramos o maior tempo de acompanhamento, segundo pesquisa, com paciente assintomático e sem sinais radiográficos de artrose glenoumeral para este perfil de fratura. Apesar do manejo de fraturas da glenoide pela artroscopia ainda estar em evolução e necessitar estudos com casuísticas e tempo de acompanhamento maiores, apresenta-se como boa alternativa de tratamento em relação à abordagem aberta, especialmente nas fraturas de menor complexidade.
